# Using Crowd-Sourced Speech Data to Study Socially Constrained Variation in Nonmodal Phonation

**DOI:** 10.3389/frai.2020.565682

**Published:** 2021-01-25

**Authors:** Ben Gittelson, Adrian Leemann, Fabian Tomaschek

**Affiliations:** ^1^ Internet Institute, Oxford University, Oxford, United Kingdom; ^2^ Center for the Study of Language and Society, University of Bern, Bern, Switzerland; ^3^ Seminar für Sprachwissenschaft, Universität Tübingen, Tübingen, Germany

**Keywords:** smartphone apps, voice quality, British English, regional variation, phonation

## Abstract

This study examines the status of nonmodal phonation (e.g. breathy and creaky voice) in British English using smartphone recordings from over 2,500 speakers. With this novel data collection method, it uncovers effects that have not been reported in past work, such as a relationship between speakers’ education and their production of nonmodal phonation. The results also confirm that previous findings on nonmodal phonation, including the greater use of creaky voice by male speakers than female speakers, extend to a much larger and more diverse sample than has been considered previously. This confirmation supports the validity of using crowd-sourced data for phonetic analyses. The acoustic correlates that were examined include fundamental frequency, H1*-H2*, cepstral peak prominence, and harmonic-to-noise ratio.

## 1 Introduction

Creaky voice—a type of nonmodal phonation resulting from the constriction of the glottis—has inspired a steady stream of frenzied editorials and news pieces in the American and British media over the past decade. *The Spectator* asked whether “creaky voice make[s] you a female yuppie—or an updated Vicki Pollard?” *The Washington Post* claimed that it hurts young women’s job prospects, and *AARP The Magazine* warned that it could damage their vocal cords. Despite this attention from the popular media, there has been little scholarly inquiry into the status of nonmodal phonation in British English since the 1980s (Henton and Bladon, 1985). While nonmodal phonation has received more attention in American English, most studies of it have relied on sample sizes of less than 50 participants and have been limited to speakers from specific geographical areas, age groups, and socioeconomic classes. This study attempts to address these gaps by investigating the use of nonmodal phonation in a diverse group of over 2,500 speakers from across the United Kingdom.

### 1.1 Phonation Types

Phonation types refer to the different methods of producing sound through the vibration of the vocal cords (Keating et al., 2015). These types can be divided into two broad categories: modal and nonmodal. In modal phonation, the vocal folds make full contact during the closed phase of the phonatory cycle; this is not the case in nonmodal phonation (Titze, 1995). Ladefoged (1971) represented phonation types as falling on a one-dimensional articulatory continuum based on the degree of glottal constriction, an assumption that underlies much of the literature on this topic (Yuasa, 2010; Keating et al., 2015; Lancia et al., 2016).

Creaky voice and breathy voice are specific types of nonmodal phonation. In this paper, the umbrella term is used when discussing multiple types of nonmodal phonation simultaneously or when the acoustic correlates in question would not allow the authors to distinguish between different kinds of nonmodal phonation. When appropriate, the more specific terms are used.

### 1.2 Acoustic Correlates of Nonmodal Phonation

**FIGURE 1:**
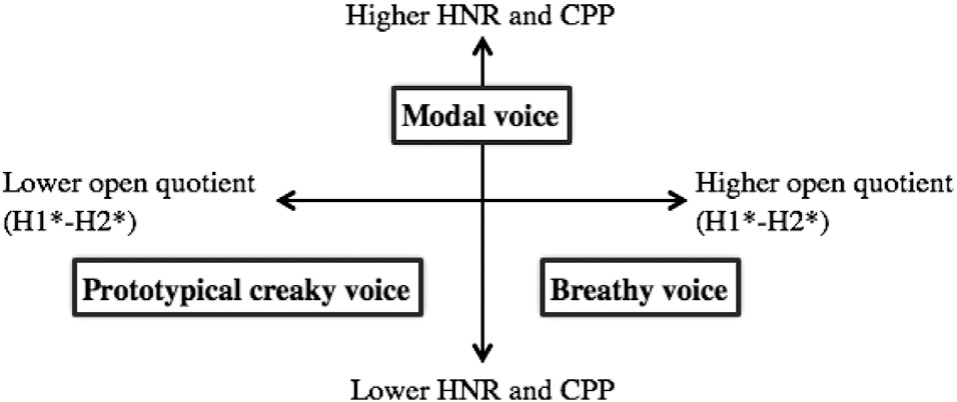
Acoustic measures of breathy and creaky nonmodal phonation. Values (higher and lower) are presented relative to those for modal phonation (adapted from Garellek (2012)).

Multiple acoustic measures may be necessary to fully describe the phonation types on this articulatory continuum, the most common of which are H1-H2 and harmonics-to-noise ratio (HNR). H1-H2 is the difference between the first and second harmonics. The first harmonic is the fundamental frequency, and the second harmonic is the first multiple of the fundamental. H1-H2 serves as a measure of spectral tilt, which is highly correlated with the degree of glottal constriction. In general, lower H1-H2 is associated with creaky voice, while higher H1-H2 occurs with breathy voice (Keating and Esposito, 2006). HNR describes the periodicity of the speech signal; nonmodal phonation results in lower HNR values than modal phonation, as the vibration of the vocal cords is usually less regular (Garellek and Seyfarth, 2016). Cepstral peak prominence (CPP), another measure of periodicity, has also been used to distinguish between modal and nonmodal phonation (Heman-Ackah et al., 2014; Garellek and Seyfarth, 2016). Heman-Ackah et al. (2014) suggested that CPP is a better measure of periodicity than HNR because it does not rely on pitch tracking and is therefore reliable even for very aperiodic signals. The relative values of H1-H2, HNR, and CPP typically associated with modal, breathy, and prototypical creaky voice are represented in Figure 1 (Garellek, 2012).

Dallaston and Docherty (2020) conducted a systematic review of studies of creaky voice in different varieties of British and American English. They suggested increasing the use of automated acoustic measurement of phonation types, as only one of the nine studies that met their inclusion criteria used this methodology. They argued that using such methods could increase the replicability and scalability of previous conclusions about the status of creaky voice in English, a gap which the present study addresses.

### 1.3 Sex Differences in the Production of Nonmodal Phonation

Previous work has found differences in the production of nonmodal phonation between men and women using read and spontaneous speech, typically with sample sizes of less than 50 participants and manual coding of phonation types. Henton and Bladon (1985) investigated sex and dialect differences in breathy voice in Received Pronunciation (RP) and Northern British English speakers’ open vowels. They selected citation forms of the open vowels /æ/, /Λ/, /ɒ/, and /ɒ/ from 61 speakers in a preexisting corpus and measured their raw H1-H2 values. The study found that British women produced breathy voice more often than their male counterparts, and that male speakers used creaky voice more frequently than female speakers. Hanson et al. (2001) examined sex differences in the production of open vowels in non-spontaneous speech as part of a larger study on models of phonation types. Specifically, they elicited the vowels /æ/, /Λ/, and /ϵ/ in carrier phrases from 21 male and 22 female participants. The authors reported two measures of spectral tilt, both corrected for the boosting effects of nearby formants: H1*-A3*, the difference between the amplitude of the first harmonic and the third formant, and H1*-A1, the difference between the amplitude of the first harmonic and the first formant. They concluded that these measures were useful for distinguishing between male and female speakers and that there was wide variation in glottal configuration for both male and female speakers.

Yuasa (2010) investigated sex differences in American English speakers’ production of creaky voice. She elicited spontaneous speech from 23 California English speakers, randomly selected 401-word samples from each one, and impressionistically coded occurrences of creaky voice. She found that women produced more creaky voice than men, a finding which was supported by Podesva (2011). However, Dallaston and Docherty (2020)—who included Yuasa (2010) in their systematic review of creaky voice in English—did not find conclusive evidence to substantiate claims of a widespread increase in the use of creak by young American women.

Garellek and Seyfarth (2016) examined acoustic differences between /t/ glottalization and phrasal creak. They used recordings of spontaneous speech from a gender-balanced corpus of 40 adults in Ohio. The researchers identified creaky phonation using preexisting annotations in the corpus and manual inspection. They concluded that linear discriminant models could be used to distinguish between different sources of creaky voice and that CPP was important for identifying this distinction.

### 1.4 Accent and Ethnicity in the Production of Nonmodal Phonation

The roles of demographic factors such as accent and ethnicity in the production of nonmodal phonation have been studied less extensively than that of sex. However, existing literature suggests that they may be related as well. Within British males, Henton and Bladon (1985) found that RP speakers creaked more than Northern British English speakers. More recently, San Segundo et al. (2019) identified instances of creaky voice in “nearly all” of the 99 Standard Southern British English speakers they studied.

Ethnicity may also play a role in nonmodal phonation. For instance, Alim (2004) linked African American identity to falsetto and “strained” voice qualities. Podesva and Callier (2015) noted that listeners could distinguish African American English speakers from white ones, even in the absence of lexical and syntactic features of African American Vernacular English. They suggested that nonmodal phonation could be responsible for this result.

In this study, we present the first large-scale acoustic analysis of nonmodal phonation for more than 2,500 speakers of British English. We examine how geography, word duration, and social factors such as sex, age, and education level affect the production of nonmodal phonation.

### 1.5 Hypotheses

We investigated the following hypotheses based on the findings described in Sections 1.1 through 1.4. The acoustic correlates used to investigate each hypothesis are described in greater detail in Section 2.5.

- Young, highly educated women creak more than men of a similar age (Yuasa, 2010; Podesva, 2011; Melvin and Clopper, 2015).
- These young, highly educated women also creak more than older men and women (Yuasa, 2010; Podesva, 2011; Melvin and Clopper, 2015).
- Men (of all ages) creak more than women (Henton and Bladon, 1985; Foulkes and Docherty, 1999).

## 2 Methods

### 2.1 Recording Method

Phonetic studies typically investigate research questions by having speakers utter words and short sentences in recording chambers at a university. Experiments under laboratory conditions allow researchers maximal control over the context of the recording and the material. However, the recording environment affects the variables of interest (Wagner et al., 2015). For example, these recording chambers do not provide the most naturalistic environment for communication, and this environment typically limits the diversity of the recorded speakers (Henrich et al., 2010; Arnett, 2016). Results are therefore biased toward the group to which scholars have access, which is typically young students. Furthermore, experiments on the university campus limit the number of participants, which ranges from five to 20 in many phonetic studies and as high as 100 or 200 on rare occasions. Small sample sizes lower the probability of detecting a true effect and raise the probability of false positives (Button et al., 2013).

With the rise of the internet, researchers can access a larger and more diverse group of participants than ever before. In addition, speakers can perform experiments in surroundings in which they feel the most comfortable. Though it requires a trade-off with potential variation in recording quality, the use of social media and private recording devices increases researchers’ ability to obtain more natural speech from a larger and more diverse group of participants.

In the present paper, we follow this argumentation. In order to record as many speakers as possible from as different backgrounds as possible, we opted to investigate phonation types not in a laboratory but rather by allowing speakers to record their voices on their own phones. To do so, we used the English Dialects App (Leemann et al., 2018), a smartphone program that allows users to record short passages in their native accents and dialects.

### 2.2 Materials

Before recording the passage, users provided data about their age, gender, education level, mobility, and ethnicity and identified their dialect by placing a pin on the locality that best corresponded to it. They then consented to the privacy agreement shown on the metadata screen. Next, participants were shown the following recording instructions: “Please record your voice in a quiet place. Hold your device approximately 6 inches/15 cm from your mouth. Please use your regional accent or dialect and speak in the way you would talk to your friends from home.” After reading these instructions, users created and uploaded recordings, in which they read a passage from “The Boy Who Cried Wolf” sentence by sentence (Deterding, 2006). The user interface then prompted speakers to self-declare their dialect by placing a pin on a map and to provide other metadata, such as age and gender. After recording, users were able to click “play” to hear their recordings and were able to re-record them if they were unsatisfied. Once satisfied, they could then navigate an interactive map where their and others’ recordings were uploaded. Upon submitting the recordings, users were shown the following notification: “by clicking ‘start recording’, you agree with our privacy policy, see info tab.” None of the information elicited–about accent, age, gender, et cetera–allows for identification of a user in the database, either individually or when considered in combination. Please see Leemann et al. (2018) for more detail about the corpus structure and demographic makeup of the speakers.

### 2.3 Speakers

Because the original data did not contain any participant identifiers, we created a participant ID using latitude, longitude, age, gender, education level and ethnicity. This yielded 2,931 participants. On that basis, we found that some participants had recorded the same stimuli more than once in different sessions. We excluded those participants (N = 159) from the analysis, leaving 2,772 speakers. We also excluded speakers from the analysis who had not yet finished school (N = 208), leaving us with a total of N = 2,564 on whom acoustic analyses were performed.

### 2.4 Signal Processing

The words used in this study were selected from the 10 sentences in “The Boy Who Cried Wolf.” Words were considered if and only if they consisted solely of vowels and voiced consonants, i.e. sonorants or phonemically voiced obstruents. The sole exception was the /h/ in “however.”

To narrow down this word list, recordings were automatically segmented using WebMAUS (Kisler et al., 2017), which aligned recordings with the corresponding sentence’s orthographic and phonemic transcription. The SAMPA phonemic transcriptions of these utterances were generated using the MAUS grapheme-to-phoneme (G2P) model and were manually verified before use. For each word, a random subset of 25 recordings was examined by hand to ensure that the forced alignments were correct. Words were only selected for analysis if at least 24 out of the 25 recordings were correctly aligned. This process led to a final list of six words in utterance-initial, medial, and final positions: “being,” “boy,” “gave,” “however,” “one,” and “while.” These words were then extracted from their respective utterances using a Praat (Boersma and Weenink, 2020) script and the TextGrids generated by WebMAUS.

We applied several methods to ensure that the extracted recordings actually contained the words of interest. We flagged words automatically on the basis of duration comparison and a calculation of zero crossing. The accuracy of the word boundaries in these recordings was then manually verified. We furthermore trimmed white spaces in an automatic procedure using the amplitude envelope as a measure of signal onset and offset. After this procedure, we analyzed all six words for 1,958 speakers, five words for 423 speakers, four words for 103 speakers, three words for 24 speakers, two words for 24 speakers, and one word for 32 speakers.

### 2.5 Data Analysis

As noted in Section 1.2, a wide variety of acoustic correlates have been used to study nonmodal phonation in previous literature. All commonly used metrics were investigated in this study to ensure comparability with prior work. These included HNR35, H1*-H2*, CPP, and F0. We used the corrected H1*-H2* rather than the raw H1-H2 measure to account for the fact that formants raise the amplitudes of nearby harmonics, making it difficult to compare H1-H2 values across different vowels (Hanson et al., 2001). This study used the correction formula described by Iseli et al. (2007) and implemented in VoiceSauce, which subtracts the amount by which the formants raise the harmonics to recover the magnitudes of the source spectrum. HNR35 is the harmonic-to-noise ratio measured between zero and 3,500 Hz. Each of the measures was calculated for 10 time steps across the word, and the mean value of those measurements was used for analysis. Numerical predictors were z-scaled to allow for comparability of the effect sizes. The following variables were used as predictors in our analyses:

- Gender (reference “female”).
- Age (mean = 34.3, sd = 14.8).
- Latitude and longitude of the location where the recording was performed. Pilot analyses revealed no effect of latitude and longitude, so these variables were omitted in the final models.
- Education level. Speakers were asked to select the degree of their education level. Possible answers were, in decreasing rank: “Higher Education (BA, BSc, MA etc., PGCE) and professional/vocational equivalents”; “A levels, Bac, vocational level 3 etc.”; “5 GCSE grade A*-C, 5 O-Levels, vocational level 2 etc.”; “Fewer than 5 GCSE grade A*-C, or fewer than 5 O-Levels,” “unknown,” and “No qualifications.” We transformed education level into a ranked scale, where higher values corresponded to higher education levels and vice versa. It is possible that education level is strongly correlated with age, posing a problem of collinearity in the model. Although the Spearman’s rank-correlation between education level and age was significant in the present study, it was not strong enough to be harmful to the regression analysis (ρ = 0.23, *p* < 0.001).
- The duration of the word. Word duration was log-transformed to reduce overly strong influence from outliers.
- Mean fundamental frequency in the extracted word (F0).

We used linear mixed-effects regression (LMER, Bates et al. (2015)) to investigate the relationship between these predictors and our measures of nonmodal phonation. We accounted for systematic effects of speakers by including random intercepts for subjects and for systematic effects of items by including random intercepts for words. Given that random intercepts shrink strong outliers more towards the mean than those already close to the mean, an estimate of *p*-values is not possible. Rather, the significance of LMER models is derived from the t-value. Absolute t-values (with t = estimate/standard error) larger than 2 are regarded to indicate a significant effect. We also included random slopes by participant. The predictors that were included as random slopes are indicated below in the Results section.

We performed an exploratory top-down and bottom-up statistical analysis, comparing different models using AIC and inspecting the significance of predictors and interactions. The final model structure included a main effect for word duration, gender, education level and an interaction between Gender and Speaker Age. In addition, F0 was used as a main effect in models fitting HNR35, H1*-H2*, and CPP.

## 3 Results

### 3.1 The F0 Measure

We tested models with three different F0 trackers: Snack (Sjölander, et al., 1998), STRAIGHT (Kawahara et al., 1998), and SHR (Sun, 2002). We used the output from each of these trackers as dependent variables and found that STRAIGHT yielded the best model fit (total AIC decrease of 5,280 between Snack and Straight, with SHR in between). We included word duration as correlated random slopes by participant.

**FIGURE 2:**
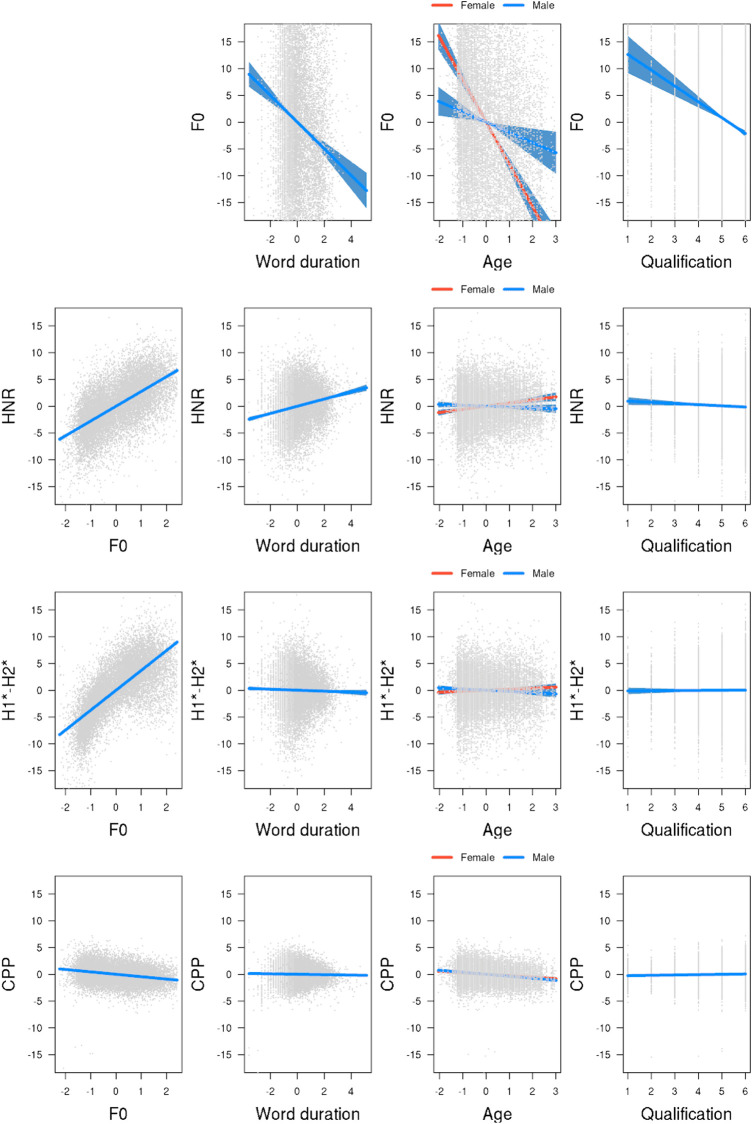
Model estimates for the four measures of modal voice. First row: F0, second row: HNR, third row: H1*-H2*, bottom row: CPP. Predictors are illustrated in the columns (adapted from Garellek (2012)).

The final model, summarized in Table 1, found lower F0 values in longer words and for male, older, and less educated speakers. The first row of Figure 2 visualizes the results, where F0 is represented on the *y*-axis and the predictor on the *x*-axis. Furthermore, the significant gender and age interaction indicates that the effect of age was smaller in male speakers than in female speakers.

**TABLE 1:** Linear mixed-effects regression summary table for F0. Absolute t-values larger than 2 are regarded to indicate significance and are highlighted in bold.

	Estimate	Std. Error	t-value
(Intercept)	221.267	7.527	**29.398**
Word duration	−2.468	0.350	−**7.073**
Gender = Male	−85.131	0.940	−**90.742**
Speaker age	−7.855	0.660	−**11.894**
Education level	−2.954	0.411	−**7.172**
Gender = Male : Speaker age	5.929	0.935	**6.341**

**TABLE 2:** Linear mixed-effects regression summary table for HNR35. Absolute t-values larger than 2 are regarded to indicate significance and are highlighted in bold.

	Estimate	Std. Error	t-value
(Intercept)	36.871	1.374	**26.823**
F0	2.661	0.096	**27.787**
Word duration	0.678	0.058	**11.777**
Gender = Male	−3.750	0.239	−**15.705**
Speaker age	0.608	0.136	**4.469**
Education level	−0.244	0.087	−**2.796**
Gender = Male : Speaker age	−0.808	0.199	−**4.068**

### 3.2 The HNR35 Measure

Testing models with HNR05, HNR15, HNR25 and HNR35 as dependent variables against each other, we found that the HNR35 measure resulted in the best model fit (AIC decrease of 7,031 between HNR05 and HNR35, with HNR15 and HNR25 in between). In addition to the predictors presented above, we included F0 as a predictor for HNR. In spite of its significant correlation with all other predictors, no effects of suppression, i.e. changes in signs, and enhancement, i.e. anti-conservative p-values, were present in this and all of the following models, which is why we regard its inclusion as safe (cf. Tomaschek et al. (2018)). We included F0 as correlated random slopes by participant. The model failed to converge with word duration as random slopes.

The second row of Figure 2 illustrates the results. We found significantly lower HNR values in words with lower mean F0, in shorter words, in male speakers relative to female speakers, and in speakers with higher education levels. The significant gender and age interaction indicates that female speakers’ HNR values increase as they get older, while this effect is reversed for male speakers. Note that the effect size is strongest for F0 and smaller by an order of 10 for all other predictors. This difference in effect size is mirrored in the other measures.

### 3.3 The H1*-H2* Measure

We included F0 as uncorrelated random slopes by participant to the model fitting H1*-H2*. The model failed to converge with word duration as random slopes. The third row of Figure 2 shows the results for H1*-H2*. We found lower H1*-H2* values in words with lower mean F0, in male speakers than in female speakers, and in longer words than in shorter words. The significant gender-age interaction indicates that older male speakers have lower H1*-H2* values. No effect of age was found for female speakers. Also, no effect was found for education level. Overall, the size of the effects is comparatively smaller for H1*-H2* than for F0 and HNR.

### 3.4 The CPP Measure

We included F0 as correlated random slopes by participant to the model fitting CPP. The model failed to converge with word duration as random slopes. The bottom row of Figure 2 displays the effects of CPP. Pitting the CPP measure against our predictors, we found significantly lower CPP values associated with higher F0 values and with older age (see Table 4). None of the other effects yielded significance.

**TABLE 3:** Linear mixed-effects regression summary table for H1*-H2*. Absolute t-values larger than 2 are regarded to indicate significance and are highlighted in bold.

	Estimate	Std. Error	t-value
(Intercept)	2.695	0.368	**7.320**
F0	4.563	0.113	**40.134**
Word duration	−0.115	0.048	−**2.415**
Gender = Male	−0.740	0.173	−**4.274**
Speaker age	0.186	0.095	1.962
Education level	−0.032	0.063	−0.507
Gender = Male : Speaker age	−0.303	0.141	−**2.140**

**TABLE 4:** Linear mixed-effects regression summary table for CPP. Absolute t-values larger than 2 are regarded to indicate significance and are highlighted in bold.

	Estimate	Std. Error	t-value
(Intercept)	19.310	0.276	**69.924**
F0	−0.468	0.040	−**11.678**
Word duration	−0.039	0.026	−1.497
Gender = Male	0.082	0.096	0.854
Speaker age	−0.277	0.052	−**5.369**
Education level	0.064	0.033	1.907
Gender = Male : Speaker age	−0.081	0.076	−1.062

## 4 Discussion

This discussion will consider the effect of demographic variables (sex, age, and education level) and F0 on the production of nonmodal phonation in British English. The results indicate that male speakers, older speakers, and more educated speakers produce more nonmodal phonation than female, younger, and less educated speakers and that more nonmodal phonation is associated with lower F0. We will end the discussion with a note on limitations.

### 4.1 Sex

Overall, our findings demonstrated that male speakers produced more creaky voice than female speakers. This was borne out in the fact that men had lower HNR than women, where lower HNR is associated with less periodicity in the speech signal and more nonmodal phonation. H1*-H2*, which measures the difference between the first and second harmonics, was also lower for men than for women, confirming that men creaked more than women. These findings are consistent with Henton and Bladon (1985) and Foulkes and Docherty (1999), who, for a subset of UK speakers, found that male speakers tended to produce more creaky voice than female speakers. In American English, two relatively recent studies (Yuasa, 2010; Podesva, 2011) demonstrated that women creaked more than men; the present results indicate that this phenomenon is not present in British English.

### 4.2 Age

Older speakers generally produced more creaky voice than younger speakers, though this effect was modulated by sex. Overall, older participants exhibited lower HNR35 and H1*-H2* values than younger ones. These findings on HNR are consistent with research on presbyphonia, or age-related changes to the vocal tract. For instance, Lortie et al. (2015) similarly found that older speakers tended to have lower HNR values than younger and middle aged ones.

Further investigation revealed that this relationship differed between sexes. For men, HNR35 and H1*-H2* followed the overall trend of decreasing with age, indicating that older men produced more creaky voice than younger ones. However, the opposite was true for women. This finding contrasts with that of Ferrand (2002), who found that elderly females had substantially lower HNR35 values than the two other age cohorts they compared to.

### 4.3 Education Level

The results for education level suggest that more educated speakers produced more creaky voice. Specifically, they exhibited both lower F0 and HNR values. Lower HNR indicates increased likelihood of nonmodal phonation—either creaky or breathy—while lower F0 suggests that the speakers produce creaky voice. These findings mirror those found for highly educated women in the U.S. (as described in Section 1.3).

Voice disorders, such as dysphonia, may also help explain this association between education level and the production of creaky voice. Roy et al. (2005) reported that the lifetime prevalence of self-reported voice disorders could be as high as 29.9 percent in the general population, while Bhattacharyya (2014) found that it was closer to 7.6 percent. Occupational voice users, such as teachers and singers, report a high prevalence of such disorders and may tend to be more highly educated (Timmermans et al., 2002). Timmermans et al. (2002) reported a statistically significant difference in acoustic measures of voice disorders between a group of occupational voice users and a control group. These measures included jitter and the highest possible F0 produced by each subject.

However, other investigations such as Niebudek-Bogusz et al. (2006) and Lehto et al. (2006) have not established a significant relationship between self reports of voice disorders in occupational voice users and objective acoustic measures of these disorders. Furthermore, the effect found in this investigation was a significant relationship between education and HNR35; this relationship was not significant for CPP, which Heman-Ackah et al. (2014) indicated was a better acoustic measure of dysphonia.

### 4.4 F0

Findings on sex and age differences in F0 align with previous research in this area, suggesting that the large-scale automated F0 tracking produced valid results. For instance, men exhibited a lower F0 than women. We also found that women’s F0 decreased with age, an effect that is consistent with research on presbyphonia (Linville and Fisher, 1985; Bruzzi et al., 2017). Lower F0 was generally associated with more nonmodal phonation, even when sex was taken into account. Lower values of H1*-H2*, HNR, and CPP, all of which indicate an increased likelihood of nonmodal phonation, were associated with lower F0. This may occur because lower frequencies of vocal fold vibration make it more likely that phonation becomes irregular, and thus creaky (Keating et al., 2015).

### 4.5 Comparison of Measures

We found that the strongest effects of gender, age and education level could be observed for F0, followed by HNR35, H1*-H2*. Our predictors showed the weakest effects for CPP. The small effect sizes for the non-F0 measures could be a result of the fact that F0 was used as a predictor in these models, accounting for a large proportion of the variance.

### 4.6 Limitations

Mobile phone recordings allowed for the development of a large and diverse data set, but this data collection method is not without its limitations. For example, European privacy regulations prohibited the collection of information about the sampling rate, bit rate, and type of encoding used by the different smartphone devices. Unknown recording conditions may have have also negatively impacted signal quality, as signals with more noise produce less reliable acoustic analyses and forced alignments. Despite a lack of a control of signal type, we still found the same patterns of phonation type variation across the United Kingdom as in previous studies that used controlled acoustic measurements. Crowd-sourced data requires a trade-off between a relative lack of control of signal quality and large, diverse data sets.

A number of studies have demonstrated that smartphone devices produce similar acoustic measurements to those found in laboratory recordings. Smartphone recordings have been shown to be sufficient for formant analysis (Decker and Nycz, 2011). Grillo et al. (2016) demonstrated that various Apple and Samsung smartphones produced similar F0, HNR, and CPP measurements to laboratory-quality microphones. A more recent study by Jannetts et al. (2019) considered four different devices (Samsung Galaxy S8+, iPhone 6s, iPhone 7, and Samsung Galaxy J3) and their effects on acoustic parameters. When compared to a reference microphone (Neumann U89i), they reported that acoustic parameters could be measured with smartphones with varying degrees of reliability. F0 and CPP, for example, provided relatively robust measures, while jitter and shimmer, which were not included in this study, did not. Jannetts et al. (2019) found that Samsung phones produced F0 values that were slightly higher than the reference measurements, while the Apple phones were slightly too low, though these errors never exceeded 2Hz. For CPP measures, all phones revealed somewhat lower values than the reference measures (Samsung c. -0.5dB; Apple -08 to -1dB). Note, though, that the authors state that these errors are so low that “their practical relevance is probably limited.” Furthermore, CPP measures did not provide significant effects in our statistical models. Unfortunately, Jannetts et al. (2019) did not study the devices’ effects on HNR parameters.

As an anonymous reviewer has pointed out, voiced plosives and glides create F0 contours (Ladd and Schmid, 2018), which will influence the HNR values. These kind of dynamic changes are inherent to the natural speech that was the focus of the current study. As a consequence, it is almost impossible to extract phonetic signals with constant F0. We therefore rather focus on a large number of samples with dynamic F0, such that any effects of dynamic transitions will be averaged across words and speakers in a large data set like the present one. Our results mirror the findings from studies that used highly controlled recording environments and measurements from vowels, which suggests that this was a valid approach.

We did not collect data on the socioeconomic or health status of our subjects due to privacy concerns, and these variables could have impacted our findings, particularly to the extent that they may be related to dysphonia. For example, Cohen et al. (2012) found that a plurality of dysphonia-related health insurance claims in the United States were filed by workers in lower paid manufacturing jobs. Dysphonia also frequently co-occurs with other health conditions, such as bronchitis and pneumonia (Cohen et al., 2012). Future studies should consider whether and how to collect such data at scale and its relationship with the production of nonmodal phonation.

## 5 Conclusion

Further research should attempt to address these concerns and consider the perceptual and phonological implications of this study’s findings. A natural progression of this work would be to conduct a perceptual study of phonation type measures. That is, do listeners perceive a difference in phonation type if words or utterances are resynthesized with different values for F0, H1*-H2*, CPP, HNR, etc.? Future studies should also consider the effect of phrase position on nonmodal phonation, as it has been suggested that creaky voice often occurs phrase-finally (Henton, 1986; Podesva and Callier, 2015).

The results of this study indicate that conclusions about the interaction of age, sex and nonmodal phonation from the 1980s and 1990s with small and geographically limited samples hold true for a large and demographically diverse group of current-day British English speakers. The use of crowd-sourced big data also allowed this study to uncover previously unobserved effects, such as a relationship between nonmodal phonation and education level. Taken as a whole, these results support the validity of using big data in phonetic studies and demonstrate that other researchers should use such data sets to confirm or challenge previous conclusions about the acoustic properties of British English speech.

## Data Availability

The data and analysis supporting the conclusions of this article can be found at https://osf.io/bvyt2/.
