# Corrigendum: Using Crowd-Sourced Speech Data to Study Socially Constrained Variation in Nonmodal Phonation

**DOI:** 10.3389/frai.2021.692064

**Published:** 2021-05-19

**Authors:** Ben Gittelson, Adrian Leemann, Fabian Tomaschek

**Affiliations:** ^1^Internet Institute, Oxford University, Oxford, United Kingdom; ^2^Center for the Study of Language and Society, University of Bern, Bern, Switzerland; ^3^Seminar für Sprachwissenschaft, Universität Tübingen, Tübingen, Germany

**Keywords:** smartphone apps, voice quality, British English, regional variation, phonation

In the original article, we neglected to include the funder “Deutsche Forschungsgemeinschaft (Research Unit FOR2373 Spoken Morphology, Project Articulation of morphologically complex words), BA 3080/3-2.”

In addition, a sentence was omitted from the **Acknowledgments** section. The section now reads:

## Acknowledgments

We thank Yang Li and Nianheng Wu, who provided insight and expertise that greatly assisted this research, although they may not agree with all of the interpretations and conclusions of this paper. We further wish to thank David Britain (Bern), Tam Blaxter (Cambridge), Marie-José Kolly (Republik), and Daniel Wanitsch (ibros.ch) for co-developing the English Dialects App. The app provided the dataset the current paper is based on.

Finally, we did not provide a link to the supporting data in the original **Data Availability Statement**. A correction has been made to the section, as seen below:

## Data Availability Statement

The data and analysis supporting the conclusions of this article can be found at https://osf.io/bvyt2/.

The authors apologize for these errors and state that this does not change the scientific conclusions of the article in any way. The original article has been updated.

